# NiO Nanoparticles for Electrochemical Insulin Detection

**DOI:** 10.3390/s21155063

**Published:** 2021-07-26

**Authors:** Jana Shepa, Ivana Šišoláková, Marek Vojtko, Libuše Trnková, Géza Nagy, Iveta Maskaľová, Andrej Oriňak, Renáta Oriňaková

**Affiliations:** 1 Department of Physical Chemistry, P.J. Šafárik University in Košice, Moyzesová 11, 040 01 Košice, Slovakia; jana.shepa@upjs.sk (J.S.); andrej.orinak@upjs.sk (A.O.); renata.orinakova@upjs.sk (R.O.); 2 Institute of Materials Research, Slovak Academy of Science, Watsonova 47, 040 01 Košice, Slovakia; mvojtko@saske.sk; 3 Department of Chemistry, Masaryk University, Kamenice 5, 625 00 Brno, Czech Republic; libuse@chemi.muni.cz; 4 Department of General and Physical Chemistry, University of Pécs, Ifjúság útja 6, 7624 Pécs, Hungary; g-nagy@gamma.ttk.pte.hu; 5 Department of Nutrition, Dietetics, and Animal Breeding, University of Veterinary Medicine and Pharmacy in Košice, Komenského 73, 041 81 Košice, Slovakia; iveta.maskalova@uvlf.sk

**Keywords:** NiO nanoparticles, insulin, electrochemical sensor

## Abstract

Diabetes mellitus represents one of the most widespread diseases in civilization nowadays. Since the costs for treating and diagnosing of diabetes represent several billions of dollars per year, a cheap, fast, and simple sensor for diabetes diagnosis is needed. Electrochemical insulin sensors can be considered as a novel approach for diabetes diagnosis. In this study, carbon electrode with electrodeposited NiO nanoparticles was selected as a suitable electrode material for insulin determination. The morphology and surface composition were studied by scanning electron microscopy (SEM), energy dispersive X-ray (EDX) spectroscopy, and X-ray photoelectron spectroscopy (XPS). For a better understanding of insulin determination on NiO-modified electrodes, the mechanism of electrochemical reaction and the kinetic parameters were studied. They were calculated from both voltammetric and amperometric measurements. The modified carbon electrode displayed a wide linear range from 600 nM to 10 µM, a low limit of detection of 19.6 nM, and a high sensitivity of 7.06 µA/µM. The electrodes were stable for 30 cycles and were able to detect insulin even in bovine blood serum. Additionally, the temperature stability of this electrode and its storage conditions were studied with appropriate outcomes. The above results show the high promise of this electrode for detecting insulin in clinical samples.

## 1. Introduction

**Figure 1:**
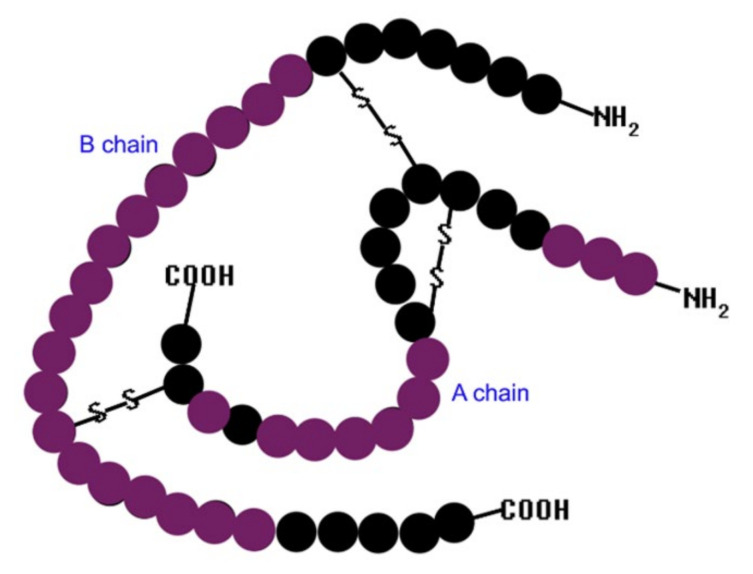
Insulin structure with the active parts of insulin in purple.

Insulin is the main hormone that regulates the glucose level in blood [1]. Insulin ensures a normal glucose level in blood, and any dysfunction in its production or action causes a serious disease called diabetes mellitus [2]. Therefore, the discovery of insulin in 1921 represents one of the greatest biomedical events of the 20th century [3]. A normal fasting insulin level in blood is 25 mIU/L (0.86 ng/L) [4]. The insulin molecule consists of 51 residues divided into the A chain (21 amino acids) and B chain (30 amino acids) linked by two disulfide bridges [5,6]. Based on another study that examined the relationship between insulin structure and activity, the active parts of insulin molecules have been identified [3]. Three areas of the A chain, at positions 1–3, 12–17, and 19, are important for the insulin structure and its function. Similarly, in the B chain, the activity of insulin provides amino acids located at positions 8–25 [3]. The insulin structure, with the active parts highlighted, is shown in Figure 1.

Since diabetes can be considered one of the most important diseases worldwide, it is essential to focus on its early diagnosis and treatment [7]. The only currently known treatment is insulin dosing [8]. Therefore, it is necessary to focus on the development of a fast, inexpensive, accurate, and reliable method for direct insulin determination. Currently, various methods like surface-enhanced Raman spectroscopy [9], microwave sensing [10], etc., are known for insulin determination. The major techniques of insulin sensing can be generally categorized into four main groups: immunoassay, chromatographic methods, optical methods, and electrochemical methods. Nevertheless, due to the many advantages of chromatographic and optical methods, they are laborious, time consuming, and high cost, and necessitate pretreatment steps without the possibility of meeting the clinical requirements criteria. Therefore, electrochemical sensors with fast response, simple preparation, and inexpensive instruments could overcome the shortcomings of these methods [11]. Various chemically modified electrodes have been suggested for promoting the oxidation and detection of trace insulin [1]. By applying a modification, it is possible to achieve a significant improvement in the analytical characteristics of bare electrodes, such as a low detection limit, high sensitivity, and wide linear range [8]. Several publications for electrochemical insulin determination are based on electrode modifications using various metal nanoparticles (Ni, Zn, Cu, and Co) [12,13,14], metal oxide nanoparticles (NiO) [15], and carbon materials [1,16], or some combination of the above. Additionally, polymer membranes such as Nafion [8,17], chitosan [18], and polyethylene glycol [19] have been widely used to prevent the fast occupation of active sites by the chloride ions in body fluids. The large benefit of the mentioned polymers is also their ability to fix nanoparticles on the electrode surface during electrochemical measurements [20].

Based on our previous research [4,20], carbon electrodes modified with a combination of multi-walled carbon nanotubes (MWCNTs), ensuring a high increase in surface area; chitosan as a polymer membrane; and NiO nanoparticles (NiONPs), which have the most pronounced catalytic activity towards insulin oxidation, proved to be the most suitable candidates for electrochemical insulin determination. Several types of carbon electrodes, such as glassy carbon electrodes (GCEs) [21], pencil graphite electrodes (PGEs) [16], and carbon paste electrodes (CPEs) [22], have been studied as potential candidates as a matrix for subsequent modification. The disadvantage of these electrodes resulting from their large size is their need for a large amount of analyte for determination. Therefore, our research is focused on the modification of screen-printed carbon electrodes (SPCE). The small size of these electrodes (L34 mm × W10 mm × H0.5 mm) leads to the minimization of the size of the working electrode (only 4 mm in diamter), the low cost, and the decrease in the analyte amount required for determination (only 50 µL), making SPCEs the most suitable candidates for the development of an electrochemical sensor for insulin diagnostics [13,20].

Herein, we aim to develop an electrochemical sensor for insulin determination based on SPCE modified by a combination of MWCNTs, chitosan, and NiONPs. NiONPs display excellent properties, such as a low cost, simple synthesis, and chemical stability. Accordingly, it is desirable to understand and investigate the mechanism of insulin oxidation on SPCE/MWCNT/NiO. Thus, three electrodes were prepared (SPCE/MWCNT/NiO0.3, SPCE/MWCNT/NiO1.0, and SPCE/MWCNT/NiO1.5) with different amounts of NiO deposited on the chitosan-MWCNT/SPCE to compare the influence of NiO loading on insulin oxidation. The morphology and elemental composition of the surface of the electrodes is examined by scanning electron microscopy (SEM) with EDX spectroscopy and X-ray photoelectron spectroscopy (XPS). Cyclic voltammetry (CV), chronoamperometry, and electrochemical impedance spectroscopy (EIS) are used to study the electrochemical properties of the prepared electrodes. All electrochemical measurements were carried out in a phosphate-buffered saline (PBS) solution simulating the presence of Cl^−^ ions at the same concentration found in body fluids. CV was also used to study the influence of interferents on the insulin oxidation of the prepared SPCE and the determination of insulin in human plasma. Finally, this study demonstrates insulin determination in real samples.

## 2. Materials and Methods

### 2.1. Chemicals and Reagents

Insulin human recombinant and phosphate-buffered saline (PBS D8662, sterile filtered) were purchased from MP Biomedicals (Irvine, CA, USA) and Biowest (Kansas City, MO, USA), respectively. Multi-walled carbon nanotubes (diameters of 3–10 nm and lengths of 1–10 µm) were purchased from BOC SCIENCES Creative Dynamics Inc. (Shirley, NY, USA). Sodium hydroxide (99%) was obtained from Milan Adamik, Laboratory Chemicals (Bratislava, Slovakia). Nickel nitrate hexahydrate (99.9%), potassium hexacyanoferrate trihydrate (99.95%), nitric acid (65%), sulfuric acid (96%), D-(+)-glucose (≥99.5%), L-ascorbic acid (99%), uric acid (≥99%), sucrose (≥99.5%), L-threonine (98%), L-tyrosine (98%), and bovine blood (Pb, Cd) were purchased from Sigma Aldrich (St. Louis, MO, USA). Insulin solutions were freshly prepared by dissolving powdered insulin in 0.1 M NaOH in PBS before every electrochemical measurement. In the same way, solutions of 0.1 mM sucrose, 0.1 mM ascorbic acid, 0.5 mM uric acid, 5 mM glucose, 83 µM tryptophan, and 78 µM tyrosine were prepared. All measurements were performed at room temperature and atmospheric pressure. Blood obtained for laboratory experiments was taken from laboratory cow. Blood used for insulin determination was allowed to coagulate for 24 h without any intervention and blood serum was obtained after clot removal. The solution was centrifuged 60 min (800 rev/min) for careful purification.

### 2.2. Instruments

All electrochemical experiments were performed via an AUTOLAB type PGSTAT302 N instrument (Metrohm, Ionenstrasse, Switzerland). SPCE type DS110 (Dropsens, Oviedo, Spain) was used for all electrochemical experiments. The used SPCEs represent a three-electrode system (working electrode = carbon electrode, auxiliary electrode = carbon electrode, and reference electrode = silver electrode) printed on a ceramic substrate (L = 34 mm × W = 10 mm × H = 0.5 mm). The structure and surface morphology of the electrodes were characterized by SEM (SEM/FIB ZEISS AURIGA, Berlin, Germany) with EDX spectroscopy (SEM/FIB ZEISS AURIGA, Berlin, Germany), and X-ray photoelectron spectroscopy (XPS) (HORIBA, Warszawa, Poland), for the surface (6–9 nm) chemical characterization, was carried out using an Axis Supra (Kratos Analytical, Manchester, UK) spectrometer. The size of NiONPs was calculated from SEM images using ImageJ software (version 4.8). To avoid differential charging of the samples, spectra were acquired with charge neutralization in the overcompensated mode. Wide spectra were acquired at a pass energy of 80 eV, and high-resolution spectra were acquired at a pass energy of 20 eV. The spectra were subsequently normalized by shifting the hydrocarbon component CHx to 285.0 eV. The atomic percentages of the elements were quantified from the high-resolution spectra of each element using CasaXPS software (version 2.3.19) after subtracting the Shirley-type background employing Gaussian–Lorentzian (G–L) peaks with a fixed G–L percentage of 30%.

### 2.3. SPCE Modification Procedure

First, MWCNTs were activated in a solution containing sulfuric acid and nitric acid at a ratio of 1:3 according to Erdélyi et al. [23]. A mixture of PBS, activated MWCNTs, and chitosan was prepared according to Šišoláková et al. [20]. Briefly, 2 mg of activated MWCNTs were dispersed in 1 mL of PBS and 1 µL of chitosan and then ultrasonicated for 1 h to obtain a homogenous suspension. Then, 10 µL of the suspension was dropwise added on the carbon working electrode surface and dried at room temperature. A solution consisting of 40 mM Ni(NO_3_)_2_.6H_2_O was used to deposit NiONPs on the chitosan-MWCNT/SPCE. The pH of the solution was adjusted to pH 2 by using nitric acid. The pulsed electrodeposition of the NiONPs on the SPCE was carried out using an optimized double-pulse sequence of potentials: E_1_ = −0.4 V (vs. Ag) and E_2_ = 0.0 V (vs. Ag). Three deposition times of NiONPs were applied to study the mechanism of insulin oxidation on SPCE/MWCNT/NiO. In all cases, the t_1_ and t_2_ times was identical with values of 0.5 s, 1 s, and 1.5 s for samples 1, 2, and 3, respectively. The prepared electrodes were activated in a 0.1 M NaOH solution via cyclic voltammetry by potential scanning between +0.1 V and +0.7 V at a scan rate of 100 mV/s for 10 cycles.

## 3. Results and Discussion

### 3.1. Scanning Electron Microscopy

**Figure 2:**
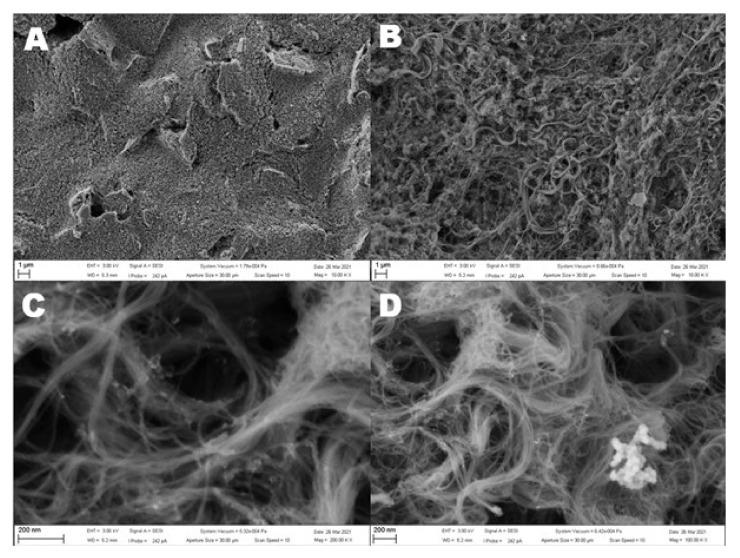
SEM images of the screen-printed electrode (**A**) and SPCE/MWCNT/NiO1.5 (**B**–**D**). (**A**,**B**) A scale of 1 µm and a magnitude of 50.00 kx. (**C**) A scale of 200 nm and a magnitude of 200 kx and 100 kx (**D**).

The SEM images of the unmodified screen-printed electrode and three modified electrodes are shown in Figure 2. As seen in Figure 2A, the bare screen-printed electrode surface is not completely smooth. After the addition of carbon nanotubes, the surface area considerably increases, as shown in Figure 2B. The carbon nanotubes create a large net of carbon material and provide an increased number of active sites, which is highly favorable for electrochemical measurement, especially in the case of protein molecules. After the electrochemical deposition of nickel oxide, clusters of nanoparticles are observed on the electrode surface. The size of the nanoparticles is approximately 40 nm. As expected, very small particles are homogeneously deposited on the electrode surface, but larger particles are observed only in the clusters.

**Figure 3:**
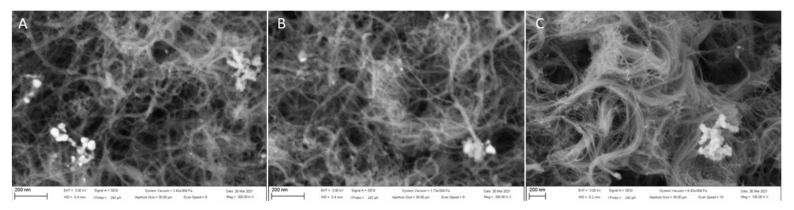
SEM images of the SPCE/MWCNT/NiO0.3 (**A**), SPCE/MWCNT/NiO1.0 (**B**), and SPCE/MWCNT/NiO1.5 (**C**). A scale of 200 nm and a magnitude of 50.00 kx.

The amount of deposited NiO particles on the electrode surface corresponds to the duration of electrochemical deposition. However, the particle size does not change with a change in deposition time duration (Figure 3). The particle size on all prepared electrodes was measured via ImageJ software and on all electrodes the particle size was in the range of 40 ± 5 nm in diameter.

**Figure 4:**
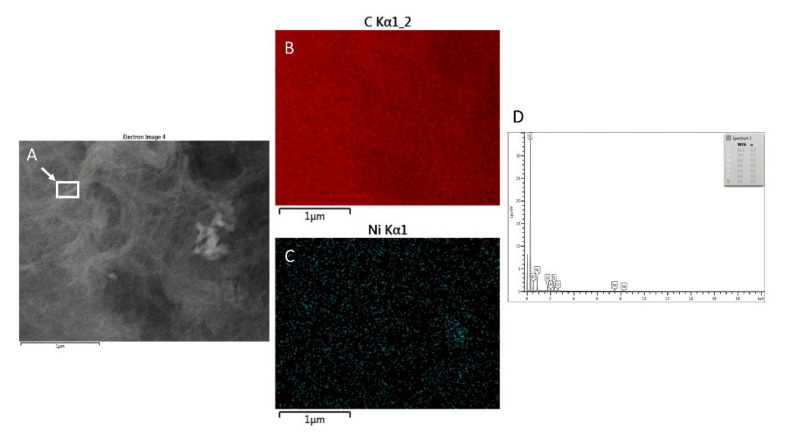
EDX analysis of SPCE/MWCNT/NiO1.5: SEM image used for the EDX analysis (**A**), distribution of carbon (**B**) and nickel (**C**) on the electrode surface, elements on the electrode surface in wt% (**D**) obtained from the marked place.

According to EDX analysis, at the location of the clusters, the amount of nickel is approximately 3.3 wt% (Figure 4B,D). The amount of oxygen on SPCE/MWCNT/NiO1.5 was 14.1 wt%, which was probably caused by the presence of NiONPs on the electrode surface. The EDX mapping image of the electrode surface displays the homogenous distribution of NiO on the electrode surface (Figure 4B), which is important for further analysis.

### 3.2. X-ray Photoelectron Spectroscopy

**Figure 5:**
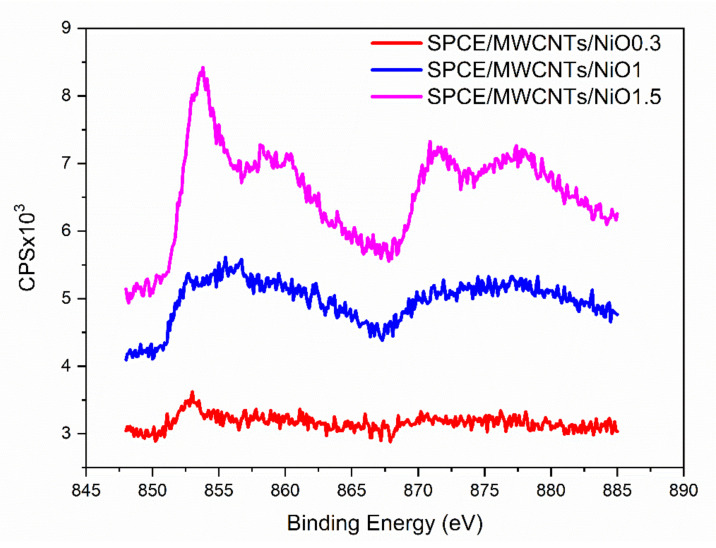
XPS spectra for the modified electrodes: SPCE/MWCNT/NiO0.3 (red curve), SPCE/MWCNT/NiO1 (blue curve), and SPCE/MWCNT/NiO1.5 (pink curve).

X-ray photoelectron spectroscopy was used to clarify the oxidation state of nickel nanoparticles deposited on the electrode surface during the two-step electrochemical deposition. High-resolution Ni 2p_2/3_ spectra were recorded as shown in Figure 5. The spectra were also compared with the nickel oxide data from the database. The Ni 2p line of the SPCE/MWCNT/NiO1.5 samples can be identified by unique multiple splitting exhibiting an intense peak at 853.8 eV. The position of NiO in the literature is reported in the range of 854–854.7 eV [24]. Based on the XPS results, the deposited nickel structures are in the nickel oxide crystal form, which is crucial information for the study of the electrochemical reaction mechanism. Moreover, it could be observed that the NiO concentration on the electrode surface increased with an increased duration of nickel oxide nanoparticle deposition.

### 3.3. Active Surface Area and Electrochemical Mechanism Study

**Figure 6:**
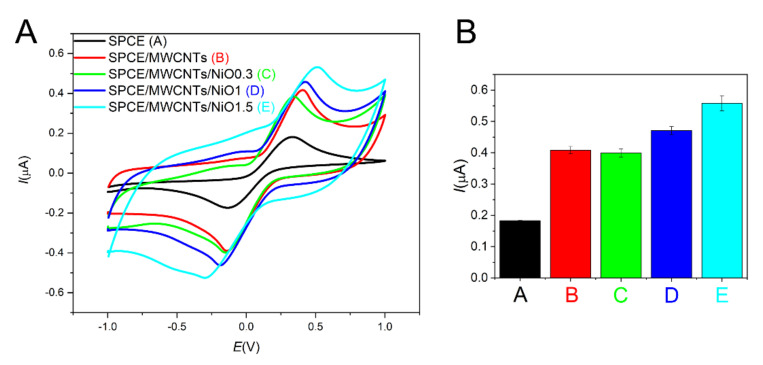
Cyclic voltammograms for different modifications of the screen-printed electrode (**A**) (SPCE—black curve **A**, SPCE/MWCNT—red curve **B**, SPCE/MWCNT/NiO0.3—green curve **C**, SPCE/MWCNT/NiO1—blue curve **D**, SPCE/MWCNT/NiO1.5—turquoise curve **E**) in 5 mM K_3_[Fe(CN)_6_]. Comparison of the current response for the particular electrode modifications (**B**).

To determine the active surface area of modified screen-printed electrodes, cyclic voltammograms in 5 mM K_3_[Fe(CN)_6_] were recorded, as shown in Figure 6. The current response increases with both the addition of multi-walled carbon nanotubes (MWCNTs) and the electrochemical deposition of NiO nanoparticles. The Randles-Ševčík equation was used for an exact calculation of the electroactive surface area [25]:

(1) $$I_{p}=0.4463.nFAC{\left(\frac{nFvD}{RT}\right)}^{1/2}$$

where *I_p_* is the current maximum in A, *n* is the number of transferred electrons, *A* is the electrode area in cm^2^, *C* is the concentration of electroactive species in mol.cm^−3^, *v* is the scan rate in V s^−1^, *D* is the diffusion coefficient in cm^2 ^s^−1^, and the rest have their typical meaning. The calculated active surface area for the unmodified screen-printed electrode is 0.23 cm^2^. With the addition of MWCNTs, the electroactive surface area increases twofold. The electroactive surface areas for SPCE/MWCNT/NiO0.3, SPCE/MWCNT/NiO1, and SPCE/MWCNT/NiO1.5 are 0.49 cm^2^, 0.58 cm^2^, and 0.69 cm^2^, respectively. Therefore, the electroactive surface area of SPCE/MWCNT/NiO1.5 increases threefold compared with that of the unmodified screen-printed electrode. The active surface area increases with increasing amounts of NiO nanoparticles, which are used as the electrocatalytic material for insulin oxidation.

**Figure 7:**
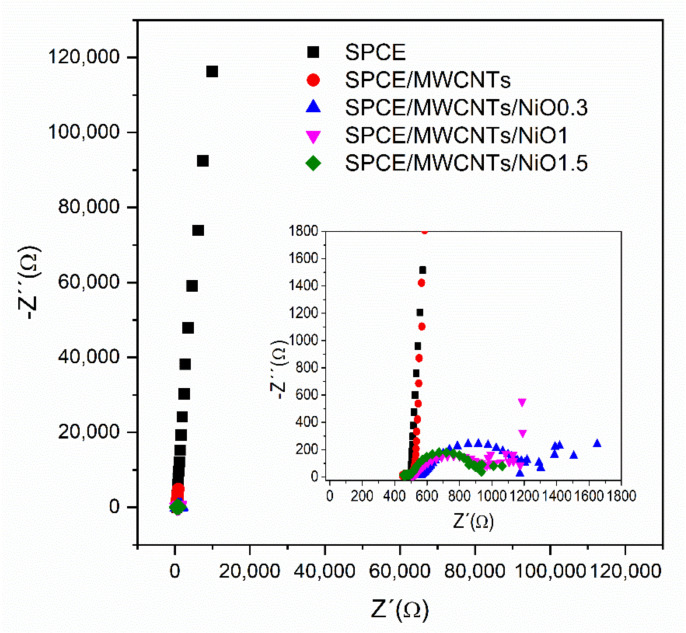
Impedance spectra at 0.7 V (vs. Ag pseudo-reference electrode) for the particular modifications of the screen-printed electrodes: SPCE—black curve, SPCE/MWCNT—red curve, SPCE/MWCNT/NiO0.3—blue curve, SPCE/MWCNT/NiO1—pink curve, SPCE/MWCNT/NiO1.5—green curve.

**Figure 8:**
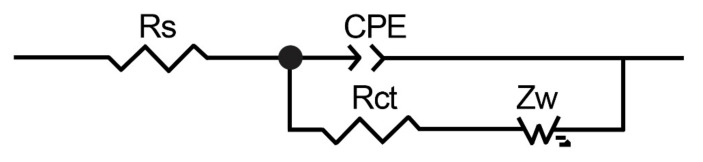
Equivalent circuit for fitting the impedance spectra.

The electrochemical behavior of modified electrodes was also studied via electrochemical impedance spectroscopy (EIS), as shown in Figure 7. Impedance spectra were recorded in 10 µM insulin diluted in a PBS solution with the addition of 0.1 M NaOH. The spectra were fitted by using the often-used equivalent circuit also called the Randles equivalent circuit, where *R_s_* is the solution resistance, *CPE* is the constant phase element, *R_ct_* is the charge transfer resistance, and *Z_w_* is the Warburg element (Figure 8). As seen in Figure 7 (Inset), the charge transfer resistance rapidly decreases after modification with NiO nanoparticles, which indicates enhanced electron transfer. The charge transfer resistance of SPCE/MWCNT/NiO1.5 is seven times lower than that of SPCE/MWCNT/NiO0.3. According to these results, SPCE/MWCNT/NiO1.5 was chosen as the best candidate for insulin determination and was used for further analysis.

**Figure 9:**
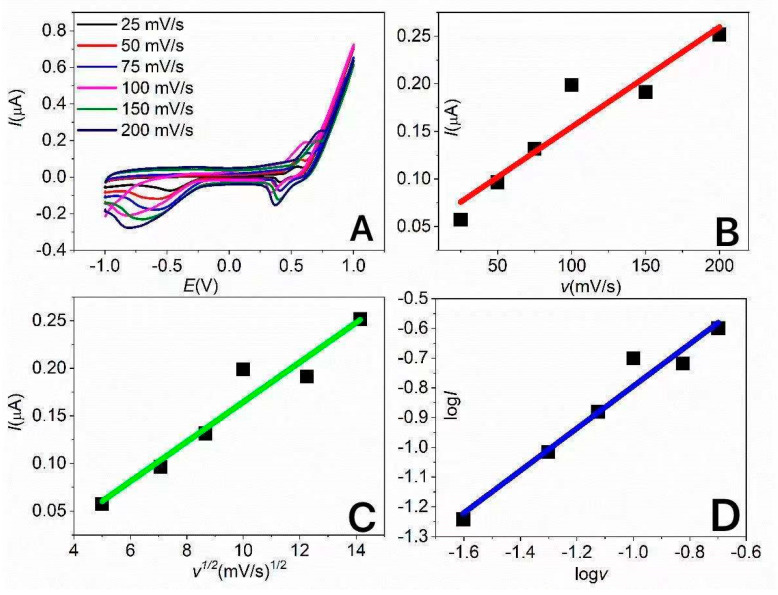
Cyclic voltammograms of 10 µM insulin on SPCE/MWCNT/NiO1.5 at different scan rates (**A**). The dependences of the peak current on scan rate (**B**), peak current on the square root of scan rate (**C**), and of logI on logv (**D**).

With the aim of studying the mechanism of electrochemical insulin oxidation, cyclic voltammograms were recorded at 50 mVs^−1^ in a 10 µM solution of insulin in 0.1 M NaOH diluted in PBS. The cyclic voltammograms of the pure electrolyte solution do not display any significant anodic or cathodic peaks. The cyclic voltammograms for the solution with added insulin show one anodic peak at approximately 0.7 V and a cathodic peak at approximately 0.37 V. These peaks could be assigned to an electrochemical reaction connected with insulin oxidation. The oxidation peaks display a higher peak area in comparison to the reduction peak, which could indicate the EC mechanism of the electrochemical reaction. In an effort to study the oxidation mechanism and determine the kinetic parameters in detail, cyclic voltammograms were recorded at different scan rates in the range from 20 mVs^−1^ to 200 mVs^−1^, as shown in Figure 9A. The rate-determining step was identified based on the dependences of the peak current on the scan rate (Figure 9B), the peak current on the square root of the scan rate (Figure 9C), and the logarithm of the peak current on the logarithm of the scan rate (Figure 9D). The dependences of the peak current on both the scan rate and the square root of the scan rate are linear, with *R*^2^ = 0.94 and *R*^2^ = 0.96, respectively. It follows that both diffusion-controlled and surface-controlled processes are possible. Additionally, in the case of the dependence of the logarithm of peak current on the logarithm of scan rate with the linear regression equation log *I* = 0.71log*v* + 0.88, the reaction could be affected by both diffusion and adsorption processes. In this case, it could be expected that electrochemical oxidation is a diffusion-controlled process because of the sluggish diffusion of insulin, as a protein with high molecular weight, to the working electrode. On the other hand, biomolecule oxidation is strongly influenced by their adsorption on the working electrode surface. Biomolecule adsorption is usually conditioned by providing enough hydroxyl groups on the surface. Therefore, the alkali solution displays a catalytic influence on the electrochemical detection of various biomolecules. Finally, in this case, both processes could strongly affect the electrochemical reaction.

As follows from previous analysis, the mechanism of insulin oxidation on modified electrodes starts with the oxidation of NiO to NiOOH, as shown in Equation (2).

(2) $$\mathrm{NiO}+{\mathrm{OH}}^{-}↔\mathrm{NiOOH}+e^{-}$$

(3) $$\mathrm{NiOOH}+\mathrm{insulin}\;↔\mathrm{Ni}{\left(\mathrm{OH}\right)}_{2}+\mathrm{product}\;$$

The second step of the oxidation mechanism occurs after the oxidation of adsorbed insulin on the electrode surface and is related to the NiOOH reduction to Ni(OH)_2_, as shown in Equation (3). When sufficient potential is applied to the electrode, NiOOH starts to be formed on the surface of the electrode. Then, the adsorbed insulin is oxidized by a chemical reaction. It could be expected that this process is a reaction with slow kinetics. Moreover, the mechanism confirms the EC mechanism, and the rate-determining step is a surface-controlled process influenced by the adsorption of insulin on NiOOH. Based on Laviron theory, the charge transfer coefficient was determined using the linear regression equation of the dependence of peak potential *E* on log*v*. For the calculation, Laviron’s equation was used:

(4) $$E=K+\frac{2.3RT}{\left(1-\alpha \right)nF}\log v$$

where *E* is the peak potential, *K* is a constant, *α* is the charge transfer coefficient, *v* is the scan rate, *n* is the number of transferred electrons, and *R*, *T*, and *F* have their typical meanings. The calculated linear regression is *E* = 0.216log*v* + 0.88, and the calculated charge transfer coefficient is 0.72, indicating an irreversible electrochemical process. The surface concentration of electroactive species (Г*_c_*) can be estimated from the dependence of the current peak on scan rate by the equation:

(5) $$I_{p}=\frac{n^{2}F^{2}vA\Gamma_{c}}{4RT}=\frac{nFQv}{4RT}$$

where *A* is the active surface area, *Q* is the peak area calculated by charges, *v* is the scan rate, and *n*, *R*, *T*, *F* have their typical meanings. Г*_c_ =* 1.54 × 0^−9^ mmol.cm^−2^ is calculated from the dependence of the peak current on the scan rate. According to the obtained results, we reason that the modified electrodes demonstrate a good synergistic effect for effective insulin adsorption.

### 3.4. Electroanalytical Properties Study

**Figure 10:**
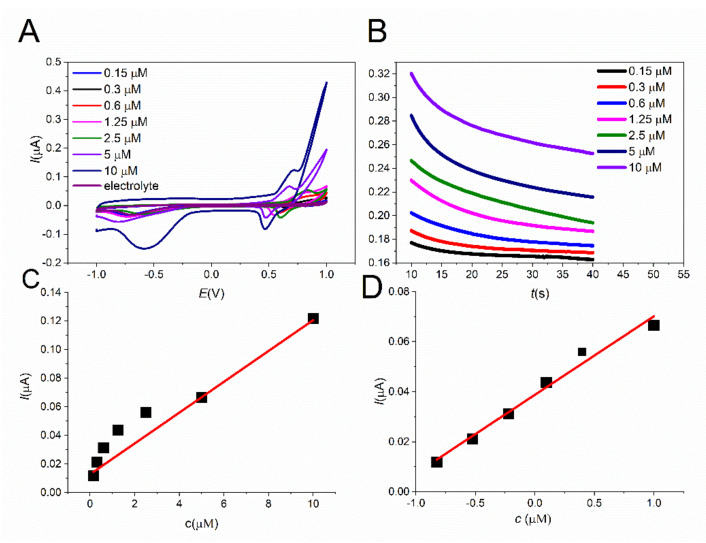
Cyclic voltammograms of different insulin concentrations on SPCE/MWCNT/NiO1.5 (**A**) at a scan rate of 50 mVs^−1^.The dependence of peak current on the insulin concentration, as fitted by a linear function (**C**). I-t responses of SPCE/MWCNT/NiO1.5 to different insulin concentrations for 30 s after applying a potential of 0.7 V (**B**). The dependence of the current response on the insulin concentration, as fitted by a linear function (**D**).

The electroanalytical properties of SPCE/MWCNT/NiO1.5 were investigated by cyclic voltammetry and chronoamperometry in PBS solution with the addition of 0.1 M NaOH and the proper amount of insulin to achieve the desired concentration at a scan rate of 50 mVs^−1^. The peak current increases linearly with the concentration of insulin, as shown in Figure 10A. Additionally, in the chronoamperometry case, the current response displays the same trend as in the previous case (Figure 10B). Both results were fitted by a linear function to assess the sensing properties of the modified electrodes, as shown in Figure 10C,D. SPCE/MWCNT/NiO1.5 displays a wide linear range from 600 nM to 10 µM, with a low detection limit of 19.6 nM and high sensitivity of 7.06 µA.µM^−1^. The electroanalytical properties of the modified electrode were compared with other electrode modifications from the literature (Table 1). As can be seen, the LOD of our prepared electrode is comparable with other electrodes mentioned in the literature [21,26,27,28], and other methods used for insulin determination [9,10]. The huge advantage of our sensor in comparison with the mentioned electrode and methods is its simple and rapid preparation, high sensitivity (7.06 µA.µM^−1^), and fast current response (less then 1 s) towards insulin oxidation determined via chronoamperometry method (Figure S1). The huge advantage of using SPCE is the small size of the working electrode (4 mm in diameter), leading to a reduced amount of analyte being needed for analysis (50 µM). 

**Figure 11:**
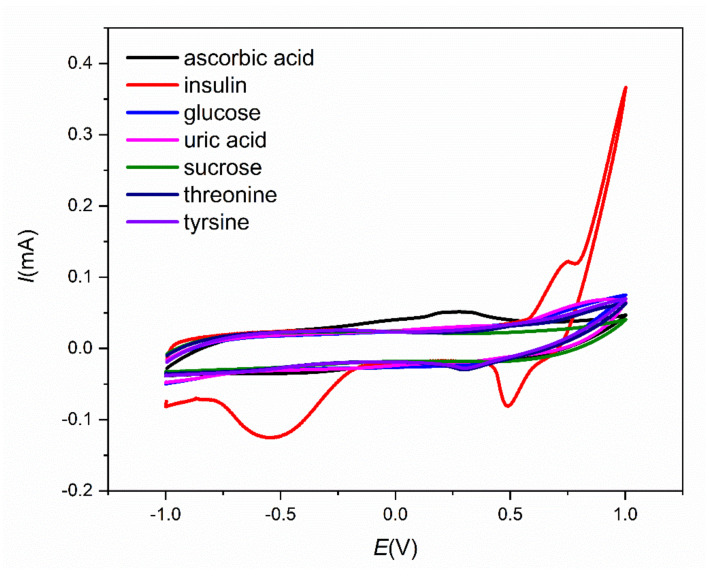
Cyclic voltammograms for the different interferents present in blood (0.1 mM ascorbic acid, 10 µM insulin, 5 mM glucose, 0.5 mM uric acid, 0.1 mM sucrose, 0.83 µM threonine, and 78 µM tyrosine).

Various electroactive species are present in blood samples, and it is necessary to study their electrochemical response under the same conditions as those tested for insulin detection. The most frequently studied species are chloride ions, ascorbic acid, uric acid, sucrose, and amino acids. The concentrations of these electroactive species were the same as those in blood samples to obtain relevant results. The cyclic voltammograms for different compounds present in blood (0.1 mM ascorbic acid, 10 µM insulin, 5 mM glucose, 0.5 mM uric acid, 0.1 mM sucrose, 83 µM threonine, and 78 µM tyrosine) are shown in Figure 11. Chloride ions and other salts that are usually contained in blood are present in the sample during all measurements because the PBS solution contains an accurate concentration of these salts to simulate blood conditions. The current responses of these interferents are much lower than the insulin current response at a potential of approximately 0.7 V. This fact creates a promising precondition for the application of the sensor in real sample testing.

**Figure 12:**
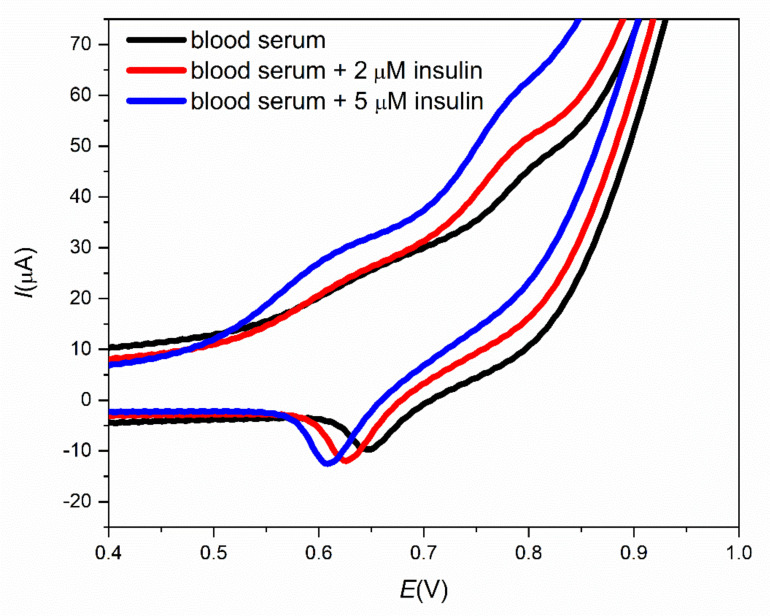
Cyclic voltammograms for the pure blood serum and blood serum samples with 2 µM and 5 µM insulin.

In an effort to verify the reliability of the modified screen-printed electrodes for routine analysis, animal blood samples were used. Pure bovine blood serum was obtained from bovine blood and used as the electrolyte in CV measurements to study the current response of a pure blood serum sample when detecting the presence of insulin (Figure 12). The presence of insulin could be declared by the oxidation peak at approximately 0.7 V, which was assigned to insulin. Moreover, with an increasing concentration of insulin in the blood serum samples, the peak current increases. According to these results, the prepared modified screen-printed electrode is a promising candidate for insulin detection in real samples.

### 3.5. Stability Study

**Figure 13:**
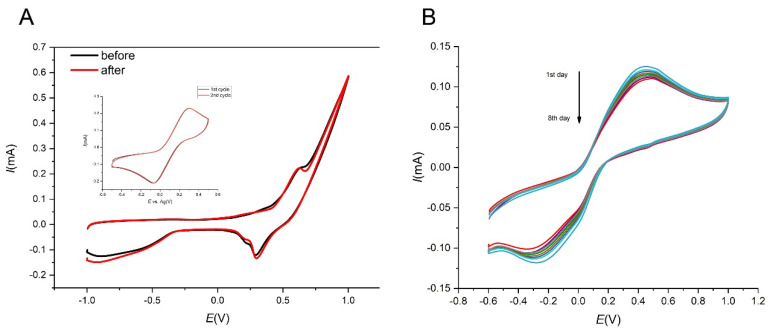
Cyclic voltammograms for 10 µM insulin in 0.1 M NaOH before and after cycling. Inset: Cyclic voltammograms to measure the stability in 5 mM K_3_[Fe(CN)_6_] for 30 cycles (**A**). Cyclic voltammograms for 5 mM K_3_[Fe(CN)_6_ obtained at 1st to 8th day of storage (**B**).

Stability is another important factor to be taken into account for real sample analysis. Stability measurements were performed in a 5 mM K_3_[Fe(CN)_6_] solution. The current response is almost constant for 30 cycles, as shown in the inset of Figure 13A. Cyclic voltammograms for insulin were performed before and after cycling with the same current value Also the long term stability of SPCE/MWCNT/NiO1.5 was studied in the 5 mM K_3_[Fe(CN)_6_] solution. The current response of SPCE/MWCNT/NiO1.5 towards insulin oxidation after 8 days decreased by only 4.1% (Figure 13B). These results show that the modified electrode is very stable over a relatively large number of measurements and after at least 8 days of storage, so we believe that this sensor could be used multiple times. This reusability could lead to material savings and is more environmentally friendly.

**Figure 14:**
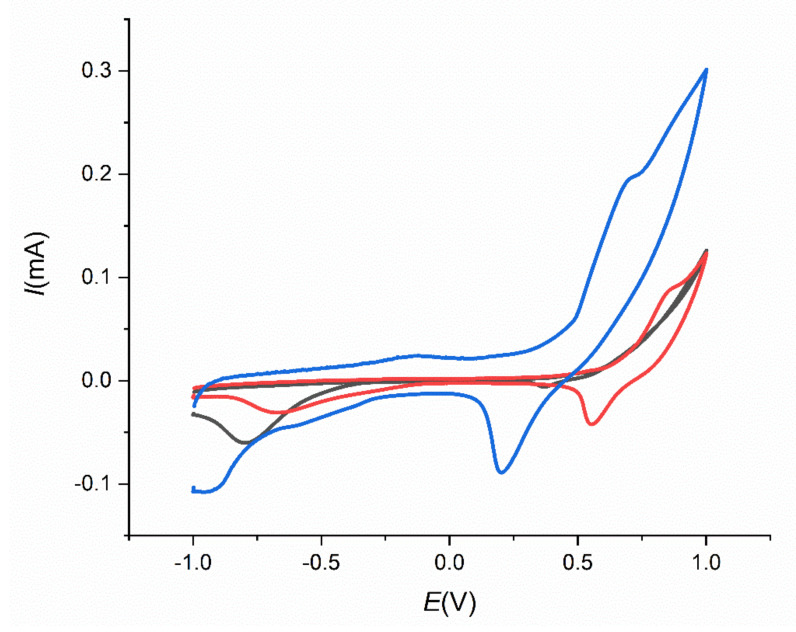
Cyclic voltammograms for 10 µM insulin in 0.1 M NaOH on SPCE/MWCNT/NiO1.5 stored for 72 h at 8 °C (blue line), 22 °C (red line), and 40 °C (black line).

Additionally, the effect of storage temperature on the stability of SPCE/MWCNT/NiO1.5 was studied. Three electrodes were prepared in the same way and stored at three different temperatures (*t*_1_ = 8 °C (Figure 14, blue line), *t*_2_ = 22 °C (Figure 14, red line), and *t*_3_ = 40 °C (Figure 14, black line) for 72 h. Figure 14 shows the cyclic voltammograms of 10 µM insulin in 0.1 M NaOH and PBS on these electrodes. As can be seen, the most suitable storage temperature for the prepared SPCE/MWCNT/NiO1.5 was 8 °C, where the visible oxidation peak for insulin was observed at potential *E* = 0.5 V. The current response towards insulin oxidation on SPCE/MWCNT/NiO1.5 decreases with increasing temperature.

### 3.6. Influence of the Temperature Study

**Figure 15:**
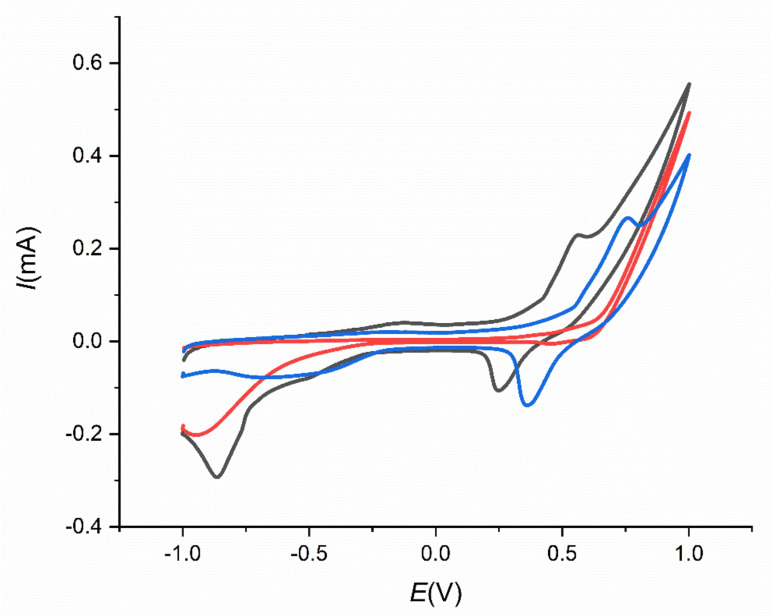
Cyclic voltammograms for 10 µM insulin in 0.1 M NaOH and PBS on SPCE/MWCNT/NiO1.5 with various temperature of the analyst’s solution, *t*_1_ = 8 °C (blue line), *t*_2_ = 20 °C (black line), and *t*_3_ = 45 °C (red line).

The effect of temperature on electrochemical determination of insulin on SPCE/MWCNT/NiO1.5 was studied to obtain the most suitable temperature for electrochemical measurements. Three electrodes modified by MWCNTs, chitosan, and NiONPs with an NiONP deposition time of 1.5 s were prepared according to the procedure described in Section 2.3. Thereafter, three solutions of 10 µM insulin in 0.1 M NaOH and PBS with various temperatures were prepared. The temperatures of the solutions were 8 °C, 20 °C, and 45 °C. Then, 50 µL of the mentioned solutions were dropped on the electrodes. The obtained cyclic voltammograms were compared and analyzed. Figure 15 shows cyclic voltammograms of three solutions with different temperature was 8 °C (blue line), 20 °C (black line), and 45 °C (red line). As can be seen, at the most suitable temperature for electrochemical measurement is laboratory temperature (20 °C), where an oxidation peak was observed for insulin oxidation at potential *E* = 0.45 V. At the higher temperature (45 °C) no oxidation peak was obtained and at the temperature of 8 °C, an oxidation peak at higher oxidation potential (*E* = 0.55 V) was observed. Based on these results the most suitable temperature of 20 °C for electrochemical determination was chosen.

## 4. Conclusions

In summary, screen-printed carbon electrodes were successfully modified by NiO nanoparticles, as evidenced via EDX and XPS analysis, as a novel tool for diabetes diagnosis. The morphology of the deposited particles was studied by scanning electron microscopy. The active surface area of the modified screen-printed electrode increased threefold, which contributed to its enhanced electrocatalytic activity. The prepared electrodes displayed very promising electroanalytical properties and good material stability after electrochemical cycling, and long-term stability. The influence of storage temperature was also studied. The most appropriate storage temperature was 8 °C. Furthermore, the modified electrodes demonstrated the ability to detect insulin in blood serum, making them promising candidates for clinical insulin detection. In our future work we would like to focus on the preparation of nickel modified electrode surface using various method to ensure a homogeneous and uniform nickel distribution on the electrode.

## Figures and Tables

**Table 1:** Comparison of the sensing properties of different insulin determination methods from the literature with SPCE/MWCNT/NiO1.5.

Electrode	Linear Range	Limit of Detection	Sensitivity	Method	Refs.
AgNF/rGO/MDEA	1 µm–1 mM	50 µM	-	Impedance spectroscopy	[19]
NiNP/CNT/CFME	2 µM–20 µM	270 nM	1.11 nAµM^−1^	Cyclic voltammetry	[26]
MWCNT/DMF/CE	250 nM–1.6 µM	-	-	Cyclic voltammetry	[29]
NiO_x_/guanine/GC	Up to 4 µM	22 pM	100.9 pApM^−1^	Cyclic voltammetry	[30]
Ni(OH)_2_ NP/Nafion-MWCNT/GC	Up to 10 µM	85 nM	5.0 AµM^−1^cm^−2^	Amperometry	[31]
IrO_x_ film electrode	0.05–0.5 µM	20 nM	35.2 nAµM^−1^	Amperometry	[32]
CHIT-CNT/GC	0.1–3 µM	30 nM	135 mAM^−1^cm^−2^	Amperometry	[33]
RuO_x_/CNT/CE	10–800 nM	-	541 nAµM^−1^	Flow-injection amperometry	[34]
Si-CPE	90–1400 pM	36 pM	107.3 pApM^−1^	Amperometry	[22]
3D gold nanoparticles pillars/ITO	100 pM–50 nM	35 pM	-	Raman spectroscopy	[9]
WGE_800_	-	35 pM	-	Whispering gallery mode	[10]
SPCE/MWCNT/ NiO1.5	600 nM–10 µM	19.6 nM	7.06 µAµM^−1^	Amperometry	This work

## Supplementary Materials

The following are available online at https://www.mdpi.com/article/10.3390/s21155063/s1, Figure S1: Current response to-wards insulin oxidation determined via chronoamperometry at potential *E* = 0.7 V.

## References

[1] Wang J., Musameh M. (2004). Electrochemical detection of trace insulin at carbon-nanotube-modified electrodes. Anal. Chim. Acta.

[2] Alberti K.G., Zimmet P.Z., Grady N.P.O., Raad I.I., Rijnders B.J.A., Sherertz R.J., David K., Supiyev A., Kossumov A., Kassenova A. (2014). NIH Public Access. Diabet. Med..

[3] Mayer J.P., Zhang F., DiMarchi R.D. (2007). Insulin Structure and Function. Mem. Fukui Prefect. Dinosaur Museum.

[4] Sisolakova I., Hovancova J., Orinakova R., Orinak A., Garcia D.R., Shylenko O., Radonak J. (2019). Comparison of Insulin Determination on NiNPs/chitosan-MWCNTs and NiONPs/chitosan-MWCNTs Modified Pencil Graphite Electrode. Electroanalysis.

[5] Rinderknecht E., Humbel R.E. (1978). Primary structure of human insulin-like growth factor II. FEBS Lett..

[6] De Meyts P. (2004). Insulin and its receptor: Structure, function and evolution. BioEssays.

[7] Duvnjak L., Blaslov K., Vučić Lovrenčić M., Knežević Ćuća J. (2016). Persons with latent autoimmune diabetes in adults express higher dipeptidyl peptidase-4 activity compared to persons with type 2 and type 1 diabetes. Diabetes Res. Clin. Pract..

[8] Arvinte A., Westermann A.C., Sesay A.M., Virtanen V. (2010). Electrocatalytic oxidation and determination of insulin at CNT-nickel-cobalt oxide modified electrode. Sens. Actuators B Chem..

[9] Cho H., Kumar S., Yang D., Vaidyanathan S., Woo K., Garcia I., Shue H.J., Yoon Y., Ferreri K., Choo H. (2018). Surface-Enhanced Raman Spectroscopy-Based Label-Free Insulin Detection at Physiological Concentrations for Analysis of Islet Performance. ACS Sens..

[10] Verma R., Daya K.S. Microwave sensing of pM concentration of insulin in buffer solution using WGM-DR. Proceedings of the 2013 Seventh International Conference on Sensing Technology (ICST).

[11] Sabu C., Henna T.K., Raphey V.R., Nivitha K.P., Pramod K. (2019). Biosensors and Bioelectronics Advanced biosensors for glucose and insulin. Biosens. Bioelectron..

[12] Yu Y., Guo M., Yuan M., Liu W., Hu J. (2016). Nickel nanoparticle-modified electrode for ultra-sensitive electrochemical detection of insulin. Biosens. Bioelectron..

[13] Šišoláková I., Hovancová J., Oriňaková R., Oriňak A., Trnková L., Třísková I., Farka Z., Pastucha M., Radoňák J. (2020). Electrochemical determination of insulin at CuNPs/chitosan-MWCNTs and CoNPs/chitosan-MWCNTs modified screen printed carbon electrodes. J. Electroanal. Chem..

[14] Li Y., Tian L., Liu L., Khan M.S., Zhao G., Fan D., Cao W., Wei Q. (2018). Dual-responsive electrochemical immunosensor for detection of insulin based on dual-functional zinc silicate spheres-palladium nanoparticles. Talanta.

[15] Rafiee B., Fakhari A.R. (2013). Electrocatalytic oxidation and determination of insulin at nickel oxide nanoparticles-multiwalled carbon nanotube modified screen printed electrode. Biosens. Bioelectron..

[16] Prasad B.B., Madhuri R., Tiwari M.P., Sharma P.S. (2010). Imprinting molecular recognition sites on multiwalled carbon nanotubes surface for electrochemical detection of insulin in real samples. Electrochim. Acta.

[17] Zarei K., Khodadadi A. (2017). Very sensitive electrochemical determination of diuron on glassy carbon electrode modified with reduced graphene oxide–gold nanoparticle–Nafion composite film. Ecotoxicol. Environ. Saf..

[18] Abazar F., Noorbakhsh A. (2020). Chitosan-carbon quantum dots as a new platform for highly sensitive insulin impedimetric aptasensor. Sens. Actuators B Chem..

[19] Yagati A.K., Choi Y., Park J., Choi J.W., Jun H.S., Cho S. (2016). Silver nanoflower-reduced graphene oxide composite based micro-disk electrode for insulin detection in serum. Biosens. Bioelectron..

[20] Šišoláková I., Hovancová J., Oriňaková R., Oriňak A., Trnková L., García D.R., Radoňak J. (2019). Influence of a polymer membrane on the electrochemical determination of insulin in nanomodified screen printed carbon electrodes. Bioelectrochemistry.

[21] Yokuş Ö.A., Kardaş F., Akyildirim O., Eren T., Atar N., Yola M.L. (2016). Sensitive voltammetric sensor based on polyoxometalate/reduced graphene oxide nanomaterial: Application to the simultaneous determination of l-tyrosine and l-tryptophan. Sens. Actuators B Chem..

[22] Jaafariasl M., Shams E., Amini M.K. (2011). Silica gel modified carbon paste electrode for electrochemical detection of insulin. Electrochim. Acta.

[23] Erdelyi B., Oriňak A., Oriňaková R., Lorinčík J., Jerigová M., Velič D., Mičušík M., Omastová M., Smith R.M., Girman V. (2017). Catalytic activity of mono and bimetallic Zn/Cu/MWCNTs catalysts for the thermocatalyzed conversion of methane to hydrogen. Appl. Surf. Sci..

[24] Weidler N., Schuch J., Knaus F., Stenner P., Hoch S., Maljusch A., Schäfer R., Kaiser B., Jaegermann W. (2017). X-ray Photoelectron Spectroscopic Investigation of Plasma-Enhanced Chemical Vapor Deposited NiOx, NiOx(OH)y, and CoNiOx(OH)y: Influence of the Chemical Composition on the Catalytic Activity for the Oxygen Evolution Reaction. J. Phys. Chem. C.

[25] Šišoláková I., Hovancová J., Chovancová F., Oriňaková R., Maskaľová I., Oriňak A., Radoňak J. (2021). Zn Nanoparticles Modified Screen Printed Carbon Electrode as a Promising Sensor for Insulin Determination. Electroanalysis.

[26] Lu L., Liang L., Xie Y., Tang K., Wan Z., Chen S. (2018). A nickel nanoparticle/carbon nanotube-modified carbon fiber microelectrode for sensitive insulin detection. J. Solid State Electrochem..

[27] Hasanzadeh M., Shadjou N., Marandi M. (2017). Graphene quantum dots functionalized by chitosan and β-cyclodextrin: An advanced nanocomposite for sensing of multi-analytes at physiological pH. J. Nanosci. Nanotechnol..

[28] Wu Y., Midinov B., White R.J. (2019). Electrochemical aptamer-based sensor for real-Time monitoring of insulin. ACS Sens..

[29] Businova P., Prasek J., Chomoucka J., Drbohlavova J., Pekarek J., Hrdy R., Hubalek J. (2012). Voltammetric sensor for direct insulin detection. Procedia Eng..

[30] Salimi A., Noorbakhash A., Sharifi E., Semnani A. (2008). Highly sensitive sensor for picomolar detection of insulin at physiological pH, using GC electrode modified with guanine and electrodeposited nickel oxide nanoparticles. Biosens. Bioelectron..

[31] Martínez-Periñán E., Revenga-Parra M., Gennari M., Pariente F., Mas-Ballesté R., Zamora F., Lorenzo E. (2016). Insulin sensor based on nanoparticle-decorated multiwalled carbon nanotubes modified electrodes. Sens. Actuators B Chem..

[32] Pikulski M., Gorski W. (2000). Iridium-based electrocatalytic systems for the determination of insulin. Anal. Chem..

[33] Zhang M., Mullens C., Gorski W. (2005). Insulin oxidation and determination at carbon electrodes. Anal. Chem..

[34] Wang J., Tangkuaram T., Loyprasert S., Vazquez-Alvarez T., Veerasai W., Kanatharana P., Thavarungkul P. (2007). Electrocatalytic detection of insulin at RuOx/carbon nanotube-modified carbon electrodes. Anal. Chim. Acta.

