# Supplementary figures and images for: NiO Nanoparticles for Electrochemical Insulin Detection
Source: Sensors (Basel). 2021 Jul 26;21(15):5063. doi: 10.3390/s21155063 (PMC8347614; doi:10.3390/s21155063)

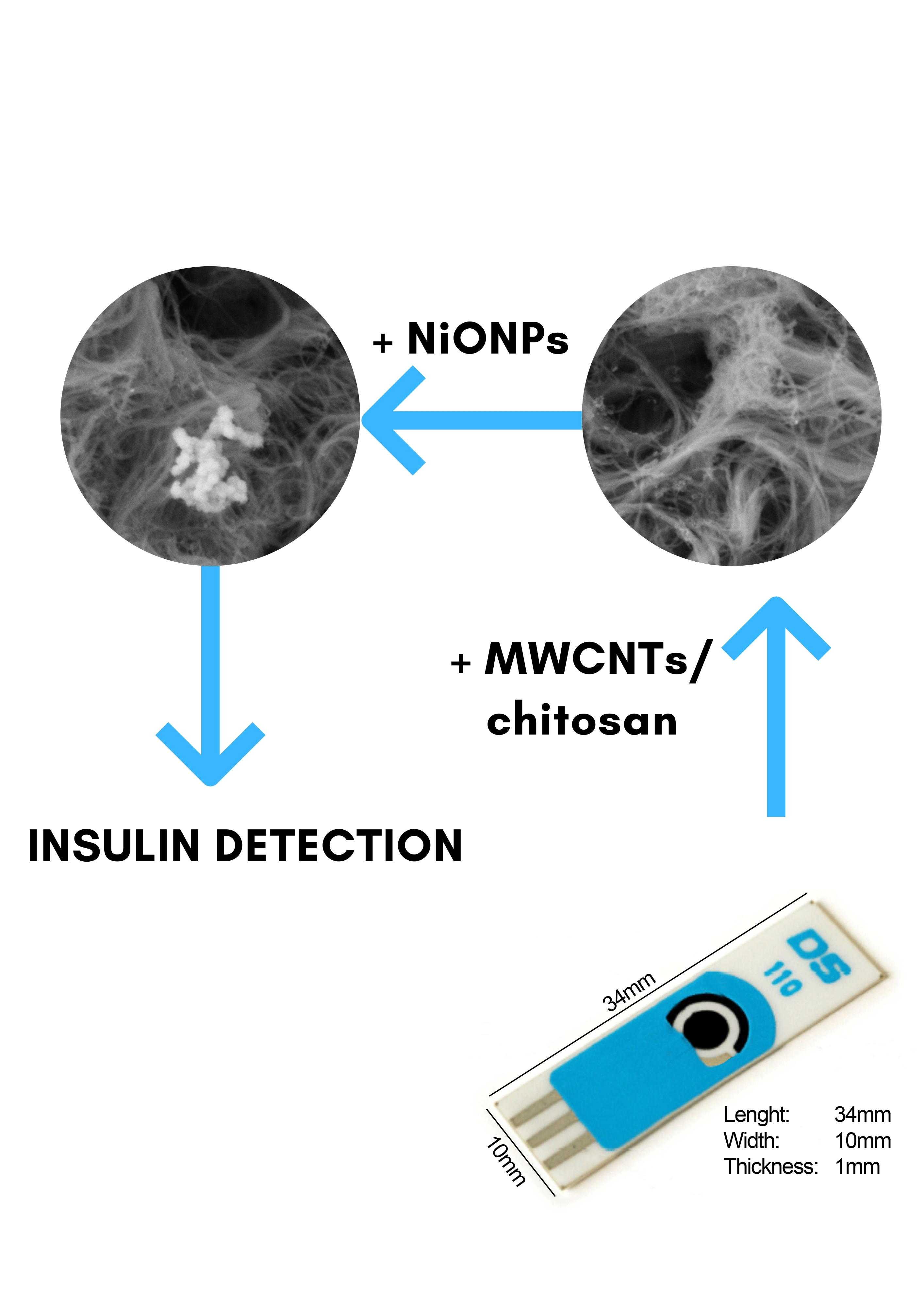

Supplement: Supplementary file 1 [file sensors-21-05063-s001.zip › sensors-1296802-supplementary.jpg]
